# Bioelectronic multifunctional bone implants: recent trends

**DOI:** 10.1186/s42234-022-00097-9

**Published:** 2022-09-21

**Authors:** Marco P. Soares dos Santos, Rodrigo M. C. Bernardo

**Affiliations:** Department of Mechanical Engineering, Centre for Mechanical Technology & Automation (TEMA), Intelligent Systems Associate Laboratory (LASI), University of Aveiro, Aveiro, Portugal

**Keywords:** Smart implants, Instrumented medical device, Implant technology, Bioelectonic implants, Biointegration

## Abstract

The concept of Instrumented Smart Implant emerged as a leading research topic that aims to revolutionize the field of orthopaedic implantology. These implants have been designed incorporating biophysical therapeutic actuation, bone-implant interface sensing, implant-clinician communication and self-powering ability. The ultimate goal is to implement revist interface, controlled by clinicians/surgeons without troubling the quotidian activities of patients. Developing such high-performance technologies is of utmost importance, as bone replacements are among the most performed surgeries worldwide and implant failure rates can still exceed 10%. In this review paper, an overview to the major breakthroughs carried out in the scope of multifunctional smart bone implants is provided. One can conclude that many challenges must be overcome to successfully develop them as revision-free implants, but their many strengths highlight a huge potential to effectively establish a new generation of high-sophisticated biodevices.

## Background

**Fig. 1:**
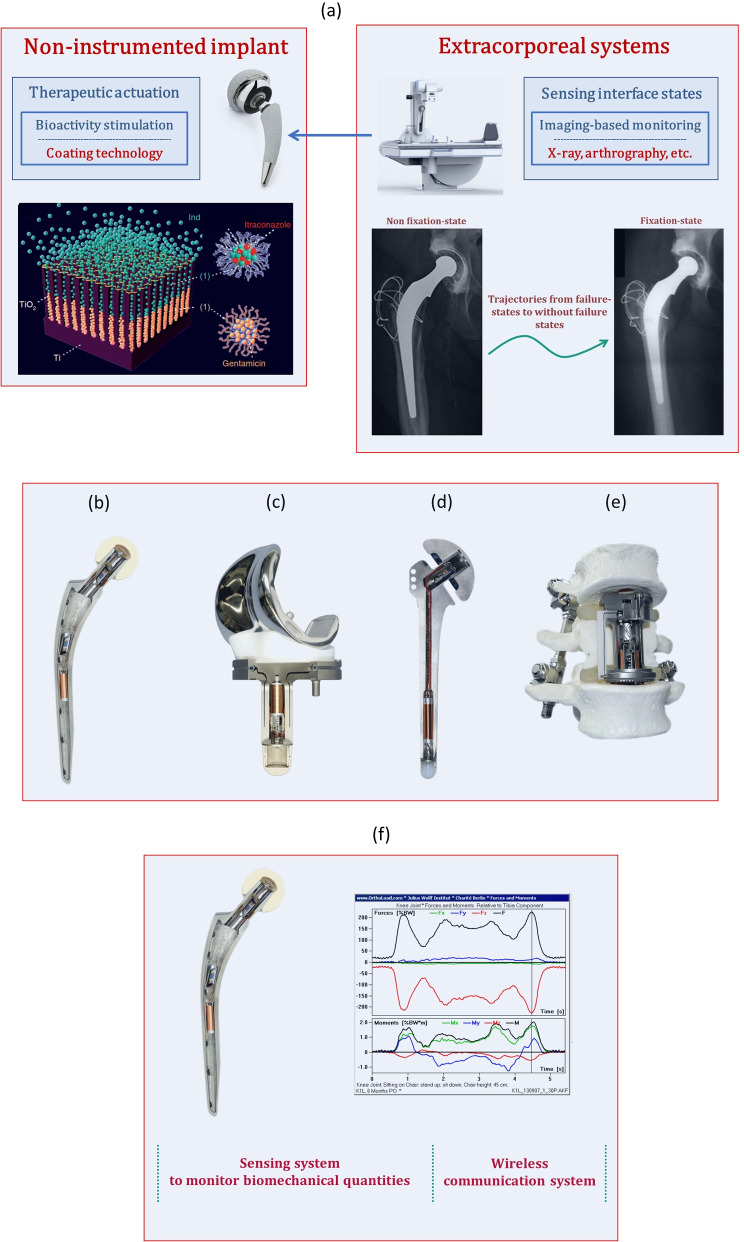
**a** Architecture used to design non-instrumented active implants. **b**-**e** Instrumented passive implants (Graichen et al. 1996, 2007; Heinlein et al. 2007; Westerho et al. 2009): hip (**b**), knee (**c**), shoulder (**d**) and spine (**e**) technologies. **f** Operations of instrumented passive implants

The development of multifunctional smart bone devices will most likely define the next technological revolution in the scope of orthopaedic implantology (Peres et al. 2022a). These are innovative bioelectronic implantable devices that have been researched to incorporate therapeutic actuation systems and bone-implant interface sensing systems (Peres et al. 2022a, b; Soares dos Santos et al. 2015). They are needed due to the limitation of current devices to significantly reduce surgical revision rates. Indeed,

1. The revision burden of the most prevalent joint replacements (THR and TKR) is currently around 10% (Ferguson et al. 2018; McGrory et al. 2016; Price et al. 2018).
2. Most patients demanding THR and TKR surgeries will be young (lower than 60 years old) in the forthcoming decades (Kurtz et al. 2009; Pabinger and Geissler 2014).
3. Stress-shielding-induced aseptic loosening remains the most common cause indicated for THR and TKR (Ferguson et al. 2018; Price et al. 2018; Soares dos Santos et al. 2014; Sumner 2015).
4. Long-term uncemented fixations are easier to achieve, since the periprosthetic interfaces with bone-implant biocontact are easier to control. A significant increasing use of uncemented fixations has been observed worldwide, but there are studies concluding that cemented implants present higher survival rates when compared to uncemented ones, mainly for patients older than 65 years old (Troelsen et al. 2013; Hailer et al. 2010). Troelsen et al. (2013) call “the uncemented paradox” to this worldwide phenomenon, highlighting the lack of consensus about which fixation method is able to achieve best performances (Green et al. 2015; Troelsen et al. 2013). The use of cemented fixations is argued with the following facts: (a) poorer performances of uncemented fixations seem to be related to the higher risks of revision of uncemented cups due to aseptic loosening (Hailer et al. 2010; b) while stress-shielding also occurs with cemented implants, uncemented fixations are more prone to induce bone loss due to this mechanical phenomenon (Sumner (2015)), although the prevalence of loosening has been reduced with the increasingly use of technological solutions based on improved geometries and bulk materials (Soares dos Santos et al. 2014; Sumner 2015; Torrão et al. 2015), as well as based on biocoatings with enhanced bioactivity (Green et al. 2015; c) initial fixation of uncemented implants is harder to establish as it requires both initial mechanical stability and effective biological response to the implant (Sumner 2015); d) fractures occur more often on uncemented stems, which increases the risk of trauma during the first postoperative year (although they may also be attributed to produced fissures during stem insertion (Hailer et al. 2010)); (e) antibiotic-impregnated bone cements have been used to reduce infection risks, but current uncemented implants are still unable to deliver drugs to the bone-implant interface, although several innovative bioactive coatings are in preclinical testing for such purpose (Goodman et al. 2013); f) better mobility and reduced post-operative pain seem to be achieved by cementing implants; (g) uncemented coatings incur increased risks of bacterial colonization, and consequent biofilm formation, due to their porous structures (Braem et al. 2015). The controversy about the higher potential of uncemented fixations to minimize implant failures is not surprising since: (i) better performances of uncemented stems have been reported (Green et al. 2015; Hailer et al. 2010; Troelsen et al. 2013). This success depends upon various factors, among which must be emphasized the use of improved bioactive coatings for uncemented stems, the increased failure risk of cemented stems (mainly the smaller sizes) and the noticed minor training of clinicians to perform successful cemented fixations (Green et al. 2015; Hailer et al. 2010; Troelsen et al. 2013); (ii) while good stabilities can be achieved by cemented fixations at short-term following arthroplasty, the mid and long-term fixation of cemented implants can deteriorate the cement-stem and/or cement-bone interfaces, increasing the risk of aseptic loosening (Sumner 2015); (iii) albeit no significant differences in surgical complications are usually found between cemented or uncemented THRs, there are significantly higher mortality risks related to cemented THRs (Hossain and Andrew 2012; McMinn et al. 2012); (iv) similar risks of revision due to infection between cemented (using antibiotic-impregnated bone cements) and uncemented THA have been reported (Hailer et al. 2010; Kapadia et al. 2016); (v) as the uncemented fixation settles a bone-implant interface, implants can be designed to promote controllable bioactivity, osteoconductivity and osteoinductivity.
5. Prior performance optimization of geometries and surface textures, to restore mobility and reduce pain, is insufficient to ensure long-term implant survival: they did not provide controllable therapeutic operations over the bone-implant interface after implant insertion (Peres et al. 2022b; Soares dos Santos et al. 2015).
6. The performance of non-instrumented active implants (Fig. 1a), those including (bio)chemical modifications of the implants’ surfaces, will hardly be the ultimate methodology to design failure-free bone implants: their ability to control the behaviour of bone-implant interfaces will not be outstanding because their delivery dynamics does not consider the bone-implant (biochemical and biomechanical) states, as well as their bioactivity, osteoconductivity and osteoinductivity cannot be changed after implant insertion (Peres et al. 2022b; Soares dos Santos et al. 2015; Sousa et al. 2021). Besides, their ability to deliver different therapeutic stimulations to target tissues in peri-implant regions will most likely be quite hard to achieve (Peres et al. 2022b; Soares dos Santos et al. 2016b, 2019; Sousa et al. 2021).

This paper highlights the recent trends related to multifunctional smart implants. Analyses were conducted considering that they are claimed as the next technological revolution in the field of orthopaedic implantology (Peres et al.^2022^).

## Smart implants

### Instrumented passive implants: the first innovation

The concept of Instrumented Implant did not firstly emerge as a disruptive concept. By designing implants embedding electronics and instrumentation, the focus was to measure biomechanical quantities (forces, moments, temperatures, deformations, etc.) in vivo (Fig. 1b-e) (Graichen et al. 2007; Soares dos Santos et al. 2014; Torrão et al. 2015). The implants’ architectures include sensing, data transfer via wireless communication, and non-autonomous powering systems (Fig. 1f), such that the in vivo data could be used to optimize biomechanical models and implant designs, as well as to perform preclinical tests and track the rehabilitation process (Peres et al. 2022b; Soares dos Santos et al. 2014; Torrão et al. 2015). Although the positive impact of these technologies is unquestionable (Haffer et al. 2022; Trepczynski et al. 2021), their performance cannot be changed after arthroplasty (Peres et al. 2022b; Sousa et al. 2021): non-optimized performances will always be observed because personalized bone-implant interations are not supported, as recently proved by Soares dos Santos et al. (2015). Indeed, this type of implants is not able to detect bone-implant interface states and to use such data to perform therapeutic actuations on peri-implant regions (Soares dos Santos et al. 2016b, 2019; Sousa et al. 2021). A personalized operation during the patients’ daily life cannot be provided. Nevertheless, as they can be engineered with smart coatings, their performance is similar to non-instrumented active implants, even though its hollow structure increases the implant fracture risk.

Although instrumented passive implants have already been used in vivo in humans (Soares dos Santos et al. 2014; Torrão et al. 2015), research has been following new paths, in particular to monitor the postoperative health state of implants, including the assessment of their postoperative translational and rotational dislocations (using inertial and piezoelectric sensing) (Almouahed et al. 2011a; Safaei et al. 2020; Tang et al. 2019), and the diagnosis of damage/fractures of implants’ components (using magnetoelastic and piezoelectric sensing) (Mouzakis et al. 2009; Safaei et al. 2017).

### Bioelectronic multifunctional smart implants: the ultimate technology

**Fig. 2:**
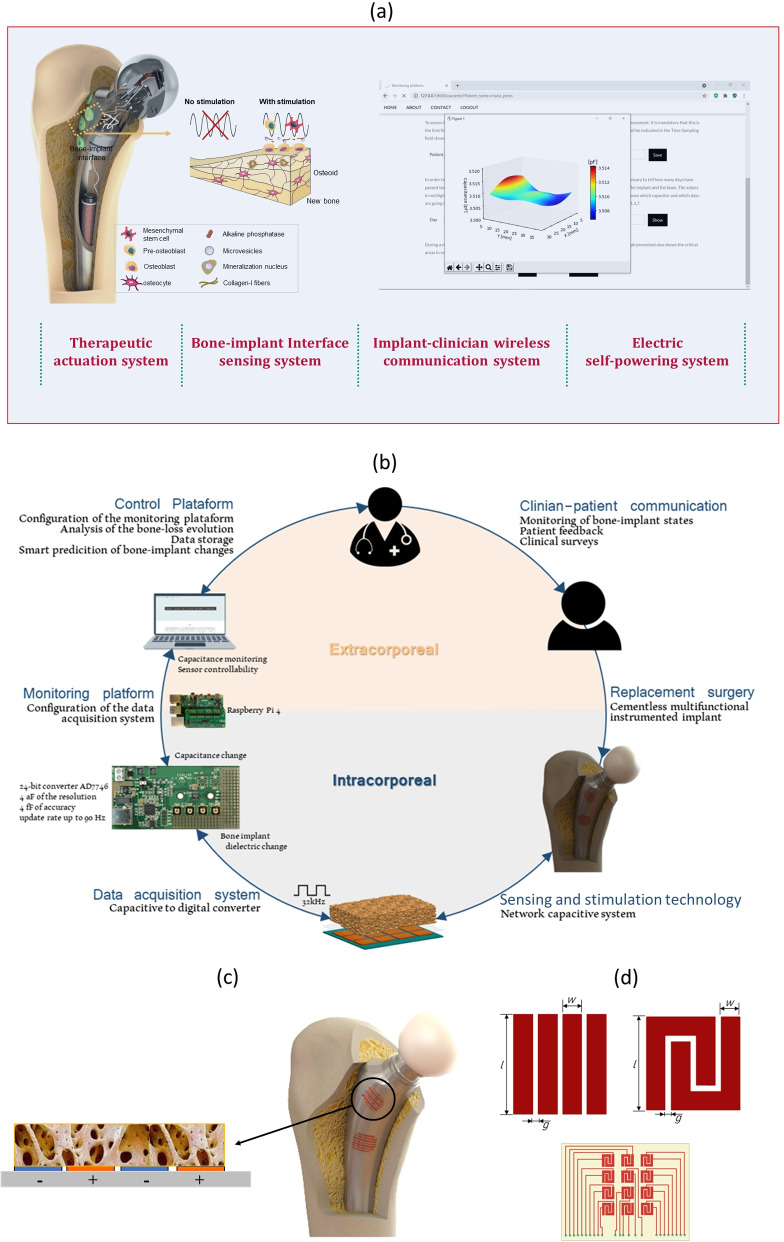
**a** Operations of instrumented smart active implants (Peres et al. 2022a; Sousa et al. 2021); **b** Master-slave distributed architecture of multifunctional smart implants (Peres et al. 2022a); **c** Co-surface capacitive stimulators incorporated within smart implants; **d** Single stripped and interdigitated electrode patterns, as well as a network stimulation design (*l*: electrode length; *w*: stripe width; *g*: gap between stripes) (Peres et al. 2022a; Soares dos Santos et al. 2016b)

Over the last decade, cutting-edge research has been conducted towards the development of instrumented active implants, such that they can operate as bioelectronic multifunctional smart implants, the ones incorporating therapeutic actuation systems, bone-implant interface sensing systems, processing systems, implant-clinician wireless communication systems and electric power systems (Fig. 2a) (Peres et al. 2022a, b; Soares dos Santos et al. 2015). Most revelant research has been carry out in four scopes: (1) therapetic actuation to promote stable bone-implant fixations; (2) sensing of the bone-implant fixation states; (3) implant-clinician data transfer; (4) self-powering of all electronics and instrumentation.

#### Implant-clinician communication systems

Data transfer operations between implants and extracorporeal systems are already well established for instrumented passive implants (Graichen et al. 1996, 2007; Haffer et al. 2022; Heinlein et al. 2007; Soares dos Santos et al. 2014; Torrão et al. 2015; Trepczynski et al. 2021; Westerho et al. 2009). Even so, three relevant innovations must be emphasized:

1. Activation circuits were already developed to activate/deactivate embedded components of the powering system (Morais et al. 2009; Morais et al. 2013).
2. High-efficient demodulation circuits for datatelemetry via inductive links (Chen et al. 2022).
3. A Master-Slave distributed architecture was implemented to establish a implant-clinician communication for personalized therapeutic actuations (Peres et al. 2022a, 2021). Using a web server hosted on Raspberry Pi, the Master system was defined as the extracorporeal system controlling all operations of the Slave system, now defined as the multifunctional smart implant (Fig. 2b).

#### Therapetic actuations on biointerfaces

The most relevant therapetic actuations have been implemented by delivering biophysical stimuli to the bone-implant interface. Mechanical stimulation by piezoelectric actuators was the first tested approach (Reis et al. 2012). Although this methodology is well supported by the encouraging biological results concerning osteogenicity, osteoconductivity and osteoinductivity (Rosa et al. 2015), no further advances were reported, mainly due to the increased risks of weakening the biointerface fixation (as their most suitable locations would be along the implants’ surface) (Soares dos Santos et al. 2016b). It is true that extracorporeal therapeutic ultrasound stimulation is widely used for bone growth and/or fracture healing of peri-implant regions (Palanisamy et al. 2022). Recent advances already succeeded to incorporate ultrasound systems inside implants, but their performances as stimulation systems have never been tested so far (Hall et al. 2021). Electromagnetic-stimulating implant systems are promising technologies towards the delivery of controllable stimuli to target regions (Peres et al. 2022a). Research on co-surface capacitive stimulation has been highlighting their suitability to be embedded in the future multifunctional bioelectric implantable medical devices. Using a non-complex and cost-effective system, with a non-parallel architecture, it is able to deliver electric field stimuli using electrodes in the same surface, regardless of the surface topology. It can comprise as many electrodes as required and according to different geometries, which can operate independently and expand to network-based architectures, allowing the delivery of electric stimuli (many controlable parameters: waveform, strength, frequency, periodicity, daily stimulation exposure, etc.) to peri-implant target areas acording to personalized stimulative therapies along the bone-implant biointerface, as defined by clinicians/surgeons throughout everyday life of patients (Fig. 2c, d) (Peres et al. 2022a; Soares dos Santos et al. 2016b, 2019; Sousa et al. 2021). Biological outcomes have been revealing their attractiveness to perform optimized trajectories of bone matrix formation and maturation and bone matrix mineralization; these conclusions were obtained by their effectiveness promoting osteogenicity, osteoconductivity and osteoinductivity for different electrode patterns (mainly the stripped and interdigitated ones) and frequency excitations (14 Hz, 1 kHz, 60 kHz, etc.) (Min et al. 2014; Soares dos Santos et al. 2016b, 2019; Sousa et al. 2021). This approach was also successfully implemented by conneting the stimulation electrodes to a coil inside an insulation layer for extracorporeal inductive powering (Zimmermann et al. 2021). Moreover, a three-electrode cathodic voltage-controlled electrical stimulator was able to significantly reduce the colony-forming units of methicillin-resistant *Staphylococcus aureus* (the main pathogen for infections associated with metallic implants) (Ehrensberger et al. 2015), which is a mandatory requirement for long-term survival of smart implants, as the periprosthetic infection is also a major consequence of implant insertion (Ferguson et al. 2018; Price et al. 2018; Soares dos Santos et al. 2014). Finally, in order to exploit the huge potential of biomagnetic stimulation of bone structures in terms of osteogenicity, osteoconductivity and osteoinductivity (Balint et al. 2013), but considering their usual requirement of very high electric current excitations (exceeding 1 A) to ensure the delivery of efficient magnetic flux densities, a small-scale *quasi-*cosurface magnetic stimulation technology requiring (up to 50-fold) lower electric current excitations was proposed to deliver personalized magnetic field stimuli to peri-implant target regions (Bernardo et al. 2019).

#### Sensing biointerface states

**Fig. 3:**
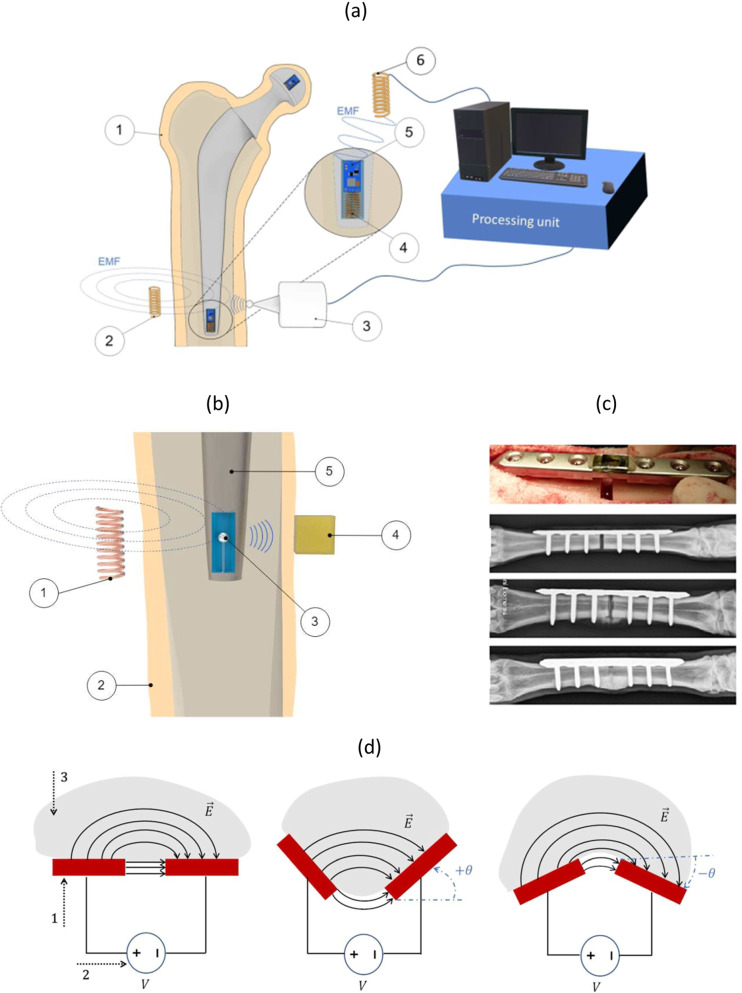
**a** Sensing technology by extracorporeal mechanical excitation and intracorporeal mechanical transduction (Cachão et al. 2020) (1 - human tissue; 2 - extracorporeal coil to power the embedded electronics through magnetic induction; 3 - extracorporeal mechanical excitation; 4 - intracorporeal coil used to power the embedded electronics; 5 - intracorporeal monitoring system; 6 - extracorporeal coil to acquire data from the sensor through magnetic induction); **b** Sensing technology by extracorporeal magnetic induction and extracorporeal mechanical transduction (Cachão et al. 2020) (1 - extracorporeal coil providing movement to the oscillator; 2 - human tissue; 3 - oscillator inside the instrumented implant; 4 - extracorporeal accelerometer that measures vibrations from the oscillator’s impact; 5 - implant); **c** Instrumented fixation plate embedding a capacitive sensing system to monitor the bone healing process (Cachão et al. 2020; d) Co-surface capacitive sensing to monitor bone-implant interface states (1 - electrodes; 2 - eletric power source; 3 - bone structures; $\theta$ - angle inclination of electrodes; $\mathbf {E}$ - electric field) (Cachão et al. 2021)

Conventional imaging methods (radiography, arthrography, scintigraphy, stereophotogrammetry, among others) are expensive, require subjective analyses and must be carried out in clinical laboratories, thus not allowing a continuous monitoring of the bone-implant interface states throughout the daily life of patients (Cachão et al. 2020). Several alternative methodologies were already proposed for instrumented implants to monitor the bone-implant fixation state: vibrometric, acoustic, magnetic induction and electric impedance (Cachão et al. 2020). Most tecnologies are based on vibrometric and acoustic methodologies, in which inertial sensores (mainly accelerometers and piezoelectric ones) are incorporated within instrumented implants to detect different bone-implant fixation states by applying (mechanical or magnetic) extracorporeal excitations (Fig. 3a) (Marschner et al. 2009). A simple ultrasonic sensor was designed for smart implants to monitor implant fixation states, intracorporeally only requiring a piezoelectric transducer and a coil (Hall et al. 2021). Another impacting technology uses extracorporeal magnetic induction to excite magnetic oscillators inside instrumented implants, such that extracorporeal mechanical transduction (using inercial sensors) can be performed (Fig. 3b) (Ruther et al. 2013a, b). An inductive proximity sensing system was proposed to detect early implant loosening, but it requires intracorporeal positioning of coils outside the instrumented implant (Mohammadbagherpoor et al. 2020). The bone healing monitoring was approached using instrumented internal fixators incorporating resistive sensing (by strain gauges), piezo-floating-gate sensing and capacitive sensing (Fig. 3c) (Borchani et al. 2016; Kienast et al. 2016; McGilvray et al. 2015). These are sensing technologies that can be customized with different geometries for non-invasive follow-up of bone-implant interface states throughout the daily life of patients. Recently, the co-surface capacitive technology was claimed to detect wide range of bone-implant fixation states, namely strong bonding, early loosening and severe loosening, including for biointerfaces coated with hydroxyapatite (Peres et al. 2022a; Soares dos Santos et al. 2021). It can provide similar advantages found in co-surface capacitive stimulators: higher controllability and personalized monitoring of target regions, as well as networked sensing. Besides, it requires very low electric currents, and can feature a hybrid sensing-acting operation (the same technology can be used both for sensing and stimulation) (Peres et al. 2022b). It is worth mentioning that a smart sensor for instrumented knee implants (ERASENSE$^{TM}$ from OrthoSensor$\circledR$) was already launched on the market (Iyengar et al. 2021). Although the technology is not described, their ability to perform early and long-term monitoring of bone-implant interface changes is reported, including infection monitoring.

#### Self-powering of smart implants

Long-term implant survival requires self-powering ability: multifunctional smart implants must incorporate high performance electric generators to simultaneously power intensive monitoring, processing, actuation and communication operations (Peres et al. 2022b; Soares dos Santos et al. 2013). Batteries are not able to ensure the power requirements of these innovative stand-alone technologies (Carneiro et al. 2020). Up to date, no effective harvesting system was engineered for smart implants, a self-powering system capable of providing suitable electric voltage-current characteristics, low maintenance costs, reduced performance losses and self-adaptability to gait patterns of patients. Current research has been focused on electromagnetic, piezoelectric and triboelectric transduction mechanisms for scavenging human motion energy to supply hip and knee implants. The most usual approach has been to use piezoelectric harvesters (Almouahed et al. 2011b, 2017; Safaei et al. 2018). These are low elecric current sources with appropriate behavior to power capacitive sensing/actuation, but with severe limitations concerning other sensing systems (e.g. the inductive ones) and processing systems, even if stacked multilayer piezoelectric elements are integrated (Lange et al. 2020, 2021a, b). Triboelectric generators are widely researched to power both large-scale and small-scale devices (Vidal et al. 2021). Although promissing results were obtained when these low elecric current sources were used to power knee implants (Ibrahim et al. 2019; Yamomo et al. 2021), long-term and stable operation of these triboelectric harvesters was not yet ensured (Xu et al. 2019). Research on high current sources has been conducted towards the development of electromagnetic harvesters with magnetic levitation architectures (Carneiro et al. 2021; Geisler et al. 2017; Soares dos Santos et al. 2016a). The most relevant approach is based on the concept of Instrumented Self-adaptive Electromagnetic Harvesting. Using a stepper motor, an accelerometer and a processing system, self-adaptability was established by changing the generators’ length as a function of human motions (Carneiro et al. 2021).

## Discussion

Before the concept of Multifunctional Smart Implant was proposed, the optimization of geometries and materials was the most common approach to enhance the performance of bone implants (Goriainov et al. 2014; Sumner 2015). Recent advances already include the design of implants with custom-made geometries with/without nanometer-scale textured surfaces to improve both primary and secondary stabilities (Benum and Aamodt 2010; Coelho et al. 2015). Several technologies were also proposed to minimize the mismatch between mechanical properties of bone and bulk materials, such as those comprising composite materials (Goriainov et al. 2014; Li and Zheng 2016), porous materials (Jing et al. 2016; Ryan et al. 2006) and multi-material structures (Simões and Marques 2005).

The design of (bio)chemical modified biosurfaces is a widely research area, nowadays regarded as the most promissing methodology to minimize the implant failure burden (Goodman et al. 2013; Goriainov et al. 2014; Navarro et al. 2008; Zhang et al. 2014). Two generations of coating materials have already emerged for non-instrumented active implants (Navarro et al. 2008). The first generation has been focused on bioactive materials, such as bioceramics, biometals and biopolymers to enhance bone-implant bioactivity and bonding (Devgan and Sidhu 2019; Navarro et al. 2008). The second generation has been directed towards biomaterials capable of promoting specific biointegration cellular responses along the bone-implant biointerfaces (Navarro et al. 2008). To enhance osseointegration and ensure non-cytotoxicity/non-genotoxicity, a wide range of coatings has been researched, including calcium phosphate-like coatings (Devgan and Sidhu 2019; Goodman et al. 2013), carbon/carbon fiber reinforced coatings (Devgan and Sidhu 2019), bioactive glass coatings (Devgan and Sidhu , 2019), bio-mimetic coatings (Devgan and Sidhu 2019), nanostructured coatings (Devgan and Sidhu 2019), anti-infection coatings (Zhang et al. 2014), biomolecule coatings (Goriainov et al. 2014; Goodman et al. 2013), drug-loaded coatings (e.g., for anti-bacterial agents delivery, growth factor delivery, anti-inflammatory and immunosuppressing drug delivery, gene therapy and nucleic acid delivery, antiresorptive drug delivery, anticancer drug delivery) (Bagherifard 2017) and multifunctional coatings (Bagherifard 2017; Raphel et al. 2016.

These are technological breakthroughs that hold potential for future implantology, but their abilities to achieve optimal performances are problematic. Indeed, an optimality analysis to passive and active implants was recently conducted using the Pontryagin Maximum Principle: conclusions highlight that optimal performances will only be obtained using some kind of sensing, actuation, communication and powering systems (Soares dos Santos et al. 2015). Therefore, the pre-optimization of geometries and textures is not sufficient, as these solutions do not provide therapeutic actuations over the bone-implant biointerfaces. Moreover, concerning the implementation of failure-free implants based on the sophistication of biosurfaces: (i) their design can be very complex, increasing as their multifunctional ability increases; (ii) their ability to perform non-biological feedback control, according to bone-implant interface states, will be an achievement quite hard to obtain; (iii) the delivery of bioactive substances, according to long-term personalized therapies, will be quite hard to achieve. These limitations can be overcome by developing non-instrumented biomaterial-based communication systems, sensors and actuators. In this scope, only electronic circuits were printed on Ti6Al4V substrates for intrinsic communication between sensors and actuators (Moura et al. 2019, 2020). Despite the huge potential impact of this research topic, fundamental science is far from being able to engineer silicon-free processing systems and electronic circuits.

The concept of Multifunctional Smart Implant emerged with a huge potential to provide superior performances for long-term implant survival. Their many strengths include:

- Strength 1 - Personalized performance: ability to provide customized therapeutic actuations throughout long time periods without disturbing the everyday life of patients, as well as the ability to enable or disable all instrumentation inside implants.
- Strength 2 - High controllability: a wide range (waveform, magnitude, frequency, periodicity, daily stimulation, etc.) of biophysical stimuli can be delivered to target peri-implant regions taken into account the bone-implant interface states.
- Strength 3 - Decision-making: performed by clinicians/surgeons or artificial intelligence algorithms.
- Strength 4 - Therapeutic and sensing abilities: (a) the same technologies can be applied both for therapeutic and sensing operations; (b) ability to be customized for different implant types and designs; (c) ability to provide therapies for several bone-implant interface conditions, including for septic and aseptic loosening.
- Strength 5 - Therapeutic complementarity: delivery of biophysical stimuli can be programmed either as the main therapeutic method or an adjuvant method.
- Strength 6 - Therapeutic simplicity: there is no need of inner reservoirs for controlled release of therapeutic drug doses (Prescott et al. 2006).

Five main challenges are expected to effectively establish Multifunctional Smart Implants as a new generation of high-sophisticated biodevices:

- Challenge 1 - Optimal biophysical stimuli must be found considering idiosyncrasies of patients.
- Challenge 2 - The interfunctional coordination between smart biophysical stimulation and smart biocoating stimulation must be implemented.
- Challenge 3 - Effective architectures of smart implants must be found: (i) design of hollowed structures minimizing fracture risks; (ii) miniaturization and encapsulation of all instrumentation inside implants.
- Challenge 4 - Smart adaptive self-powering systems must be designed considering time-varying body motion dynamics.
- Challenge 5 - Autonomous operation must be ensured, which may require the design of artificial intelligence algorithms for therapeutic decision-making.
- Challenge 6 - Cost-effective technological solutions must be ensured.

**Fig. 4:**
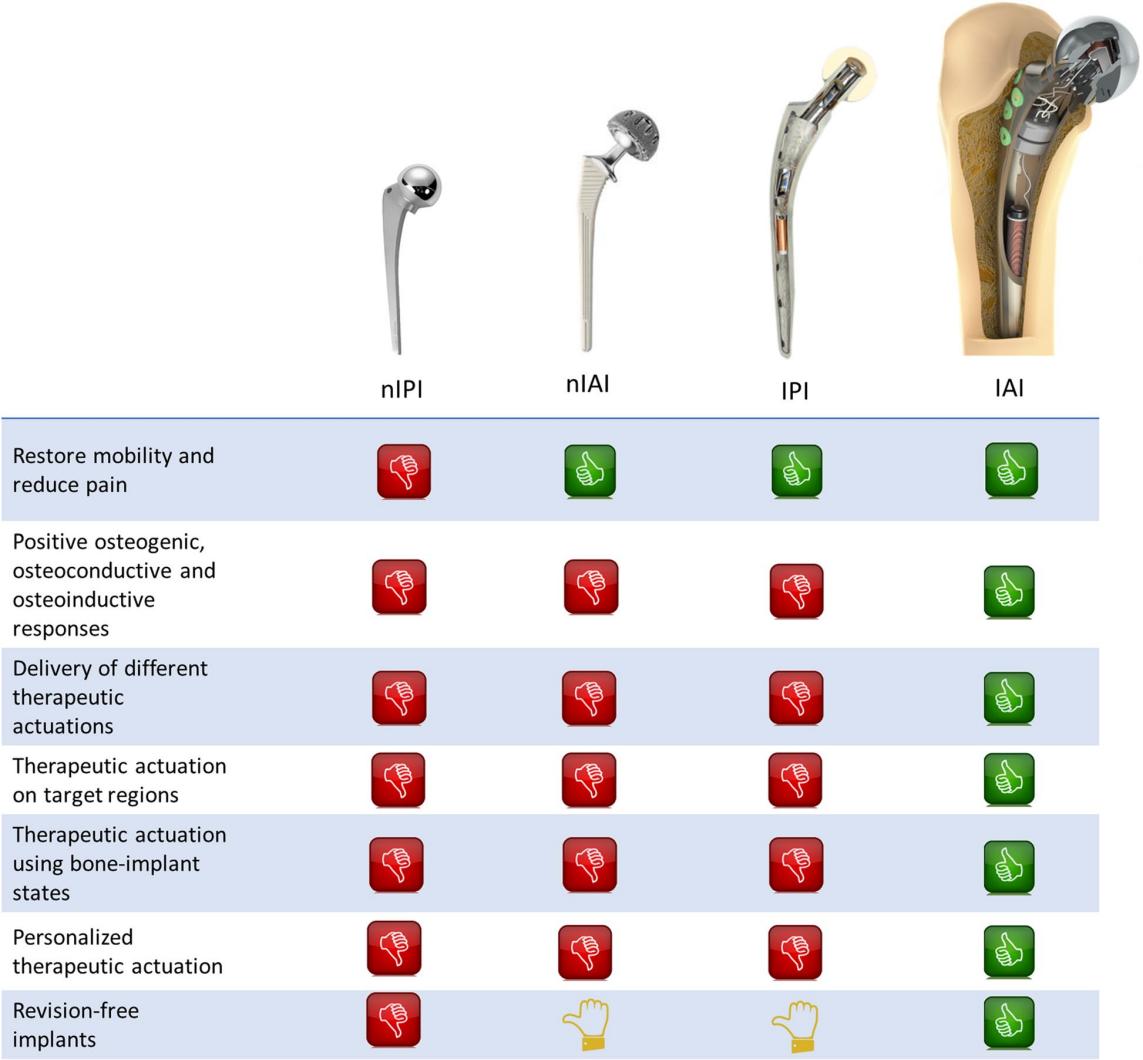
Comparative analysis to the performance of non-instrumented passive implants (nIPI), non-instrumented active implants (nIAI), instrumented passive implants (IPI) and instrumented active implants (IAI)

Figure 4 provides a comparative analysis to the performance of the four implant technologies (non-instrumented passive implants, non-instrumented active implants, instrumented passive implants and instrumented active implants), considering their potential to deliver optimized therapeutics on biointerfaces (notice that a closed-loop feedback of sensing data is mandatory to ensure optimality (Soares dos Santos et al. 2015)). One cannot state that non-instrumented active implants are unable to obtain optimized performances: one can only emphasize that the controllability of the bone-implant interface behaviour will most likely be quite hard to implement using this type of implants. Despite multifunctional smart implants will be more expensive than non-instrumented implants, the number of years lived with disability will most likely be minimized and, therefore, the societal burden will be significantly reduced.

## Conclusions

Multifunctional smart implants are promissing technologies to revolutionize the field of orthopaedic implantology. Indeed, their strengths highlight their huge potential to ensure long-term implant survival if they are successfully developed (Peres et al. 2022b; Soares dos Santos et al. 2015): (1) ability to perform personalized and controlled therapeutic stimulations without disturbing the everyday life of patients (fulfilled criteria: customized performance and high controllability); (2) sensing and therapeutic atuations can be performed by clinicians/surgeons or artificial intelligence algorithms (fulfilled criterion: decision-making capability); (3) there is at least one technology (co-surface capacitive system) with ability to ensure both therapeutic and sensing operations in different implant types and designs (fulfilled criterion: multi-operationality); (4) therapeutic actuation can be defined either as the main therapeutic method or just an adjuvant one (fulfilled criterion: therapeutic complementarity).

The full development of multifunctional smart implants still requires significant research efforts, mainly the identification of the optimal biophysical stimuli for personalized control of biointerface states, and the development of smart self-powering systems for adaptive operation according to body motion dynamics and required voltage-current characteristics. The miniaturization and encapsulation of all instrumentation inside implants will be challenges that must be faced, as well as the design of hollowed structures minimizing fracture risks. Their ability to provide long-term autonomous operation is also mandatory. Finally, in vivo experimental tests in animal models and humans must be carried out for translational research. Indeed, up to date, no clinical trials were carried out using multifunctional instrumented active implants).

## Data Availability

Not applicable.

## Abbreviations

THR: Total Hip Replacement
TKR: Total Knee Replacement
